# Early loading of hydrophilic titanium implants inserted in low-mineralized (D3 and D4) bone: one year results of a prospective clinical trial

**DOI:** 10.1186/1746-160X-9-37

**Published:** 2013-12-09

**Authors:** Uwe Held, Dennis Rohner, Daniel Rothamel

**Affiliations:** 1 cfc Hirslanden Medical Center, Aarau, Switzerland; 2 Department of Oral and Maxillofacial Plastic Surgery, University Hospital of Cologne, 50924 Cologne, Germany

**Keywords:** Titanium implants, Hydrophilic surface, Healing time, Bone quality, Weak bone

## Abstract

**Introduction:**

Pure titanium is the material of choice for contemporary dental implants. However, superficial reaction of the moderately rough titanium surface with atmospheric components decreases its hydrophilicity. INICELL® represents a chemical alteration and hydrophilization of a moderately rough i. e. sand-blasted and acid-etched titanium surface. The hydrophilicity leads to a more homogenous adsorption of proteins on the implant surface in-vitro, supporting the activation of a higher number of platelets and the generation of a homogenous, complete fibrin matrix in the early phases of osseointegration. This in turn helps to reduce the healing time and enhances the predictability of osseointegration in compromised bony situations.

The objective of this case series trial was therefore to investigate if early loading (after 8 weeks) of hydrophilic INICELL implants is feasible in patients with reduced bone quality.

**Methods:**

In 10 patients, 35 hydrophilic implants were placed in sites revealing bone quality class 3 and 4, and uncovered after 4 weeks. Eight weeks later implants were released for loading if the tactile resistance was ≥35 Ncm. Lower resistances resulted in 12 weeks initial healing period. Insertion torque, ISQ, tactile resistance and vertical bone level were evaluated at implant installation, after 4 weeks (uncovering), 8 or 12 weeks (loading), and 12 weeks and one year after loading.

**Results:**

Mean implant insertion torque was 21 Ncm. 31 (88.6%) showed a tactile resistance of >35 Ncm after eight weeks and were released for prosthetic loading. Eight weeks after insertion, one implant (2.9%) had to be removed following a soft tissue complication. One implant had to be removed after 4 weeks due to a technical complication (fractured Osstell-abutment), it was therefore excluded from the analysis.

33 of 34 implants (97%) were loaded to occlusion and were in situ/functional one year after implantation. ISQs increased from 43 at baseline to 63 at eight weeks, and 72 at three months after loading. Then, ISQ remained constant until one year after loading.

**Conclusions:**

Within the limitations of this prospective case series, hydrophilic implants may allow for shortening of the initial healing period even in bone with compromised density.

## Introduction

In dental implantology, bone quality and quantity have a substantial influence on the primary implant stability, substantially influencing the osseointegration success of implants. Primary implant stability is defined as biomechanical stability that is achieved immediately after implant insertion. In order to increase the primary stability of implants, modifications of the implant geometry and surface texture were introduced. In addition, efforts have been undertaken to shorten the healing phase after implant surgery by increasing the surface hydrophilicity.

After dental implant insertion into the prepared bed, areas with direct physical contact of bone matrix to the implant surface and areas without direct bone contact can be distinguished. The voids between bone and implant surface are filled with serum and blood clots. Absorption of proteins and macromolecules occurs on the implant surface. This conditioning film will allow the fixation of fibrin fibers. The formation of a fibrinogen-based matrix enables the infiltration of cellular components. The opened medullar spaces release cytokines, growth factors, as well as mesenchymal cells with osteogenic potency. This initiates the formation of new bone matrix [1,2].

The time span between the blood coagulum reorganization and the formation of a new periimplant bone matrix is approx. four weeks. This time is the critical implant healing period. In this phase the mechanical implant stability has to be transformed into a biological anchorage, nevertheless the implant stability may be at lowest during this transition. After the critical period of implant healing the bone apposition continues gradually with an increasing secondary implant stability [3,4].

Faster formation of a periimplant bone matrix leads to an earlier increase of the secondary stability and in turn to shorter osseointegration periods. The application of growth factors has been considered in their capacity to speed up osteogenic processes. While bioactive, mitogenic molecules such as PDGF (platelet-derived growth factor, transforming growth factor-β2) induce an accelerated division of fibroblasts and osteoblasts. BMPs (bone morphogenetic proteins) influence the mesenchymal cells in a morphogenic way so that osteogenic determination of mesenchymal cells will take place [5-7]. Moreover, an additional direct influence on new bone generation can be induced by specific chemical properties of the implant surface [8]. Ionized, loaded surfaces have a higher surface energy than non-ionized surfaces and therefore they offer improved hydrophilic properties that stimulate the biological interaction between the implant and its environment [9].

Hydroxylation of a titanium surface leads to an improvement of its ionization properties and increases its hydrophilicity. This enables an improved adsorption of proteins on the surface [3]. The enhanced differentiation of pre-osteoblasts and subsequent generation of new bone speeds up the osseointegration process [10,11], which in turn can be used to reduce the healing period before implant loading is possible [12,13].

Thanks to its ideal mechanical and chemical properties, grade 4 titanium is the material of choice for the majority of dental implants. However, superficial reaction of the titanium surface with atmospheric components decreases the hydrophilicity of titanium implants. The INICELL® protocol describes a chemical alteration and hydrophilization of titanium implants showing a sand-blasted and acid-etched surface. Its hydrophilic properties lead to a more homogenous adsorption of proteins on the implant surface in-vitro [14], supporting the activation of a higher number of platelets and the generation of a homogenous, complete fibrin matrix in the early phases of osseointegration [15]. Animal studies have shown that this leads to a faster bone apposition on the implant surface [16,17], which in turn may reduce the healing time and enhance the predictability of osseointegration in compromised bony situations.

The objective of this prospective clinical case series was therefore to investigate if early loading (after 8 weeks) of hydrophilic INICELL implants is feasible in patients with reduced bone quality. As primary outcome, implant survival rate and the proportion of patients released for loading 8 weeks after implant placement, was calculated. Insertion torque, tactile resistance and ISQ changes were recorded as supportive measurements.

## Materials and methods

### Study conduct

Ten partially edentulous patients (age: 18–75 years) that needed tooth replacement in areas of bone quality class 3 and 4 were included in this single group, prospective case series study. The follow-up period was one year. The trial was conducted in compliance with the ethical requirements of the Helsinki Declaration. The study protocol was approved by the Ethics Review Board of Canton Aarau (CH).

In the time period between January and November 2009, a total of 36 implants with a novel, chemically modified surface were placed. 35 implants have been inserted in areas with reduced bone quality of ten patients (in one patient 1 of 2 implants was inserted in a site with good quality bone) in the Cranio-Facial Center Hirslanden (Aarau, CH). The bone quality in these regions had to be Class 3 and 4 [18] corresponding to D3 and D4 [19]. Bone quality diagnosis was established on CBCT scans that were taken in progress of implant planning. The study exclusion criteria included strong nicotine abuse (>10 cigarettes/day), bruxism, radiation therapy earlier than one year before implantation, as well as discontinued radiation therapy and any diseases that could have negative influence on osseointegration. In addition, patients with non-compensated diabetes, chronic infectious and other metabolic diseases were excluded.

Implants were placed after performing anamnestic, as well as clinical and radiological examinations. These included an orthopantomogram (OPG) and cone beam computed tomography (CBCT). Used implants had cylindrical design with self-tapping threads and hydrophilic enossal surface RC (ELEMENT RC INICELL), Thommen Medical AG, Waldenburg, CH). Surface hydrophilization was done chairside immediately before implant insertion. Hereby, sterile implants were conditioned using a diluted sodium hydroxide solution according to the manufacturer´s instructions.

All patients underwent a two-stage procedure. Implant bed preparation was performed in accordance to the manufacturer´s protocol. 36 Thommen ELEMENT RC implants (platform Ø 4.0, 4.5 und 5.0 mm, lengths 8.0, 9.5, 11.5, 12.0 and 14.0 mm) were placed epicrestally, 35 of them into bone quality D3 and D4. In one patient 1 of 2 implants was inserted in a site with D2-bone, it was therefore not included in the subsequent evaluation. For each of the 35 implants, the maximal insertion torque was recorded. Implant stability quotient (ISQ) was measured using a contact-free device (Osstell®, Ostell AB, Göteborg, Sweden). Immediately after the surgery a cone beam scan (CBCT, Newtom VG, Verona, Italy) was performed. For post-surgical infection prophylaxis 300 mg Clindamycin was prescribed three times a day. Patients were instructed to rinse with a 0.1% chlorhexidine solution 3 times daily over a period of one week and eat soft diet for three months. Mefenamic acid was used for analgesia.

All implants were uncovered four weeks after insertion. Using a calibrated parodontal probe the distance between implant shoulder to the marginal bone level was measured mesially, buccaly, distally and orally. ISQ measurements were repeated, and tactile resistance was measured. This follow-up test of implant stability was done using the MONO® torque wrench (Thommen Medical AG, Waldenburg, CH). A careful assessment of torque momentum was done in the “OUT” position until the appearance of a first sign of rotation or pain, up to a maximal torque of 35 Ncm.

Eight weeks after implant installation ISQ, bone measurement and implant stability tests were repeated. Implants that showed a tactile resistance of ≥35 Ncm were released for prosthetic loading. Implants that did not achieve the tactile resistance of ≥35 Ncm were followed up after additional 4 weeks, using the same protocol. If they had achieved the minimum specified tactile resistance level mentioned above, they were released for loading.

All implants were then followed up 12 weeks and one year after loading. At this time point, tactile resistance, ISQ and pocket depths were evaluated. Patient data were entered into a dedicated database. Descriptive statistics was calculated using commercially available software (Excel). This was an exploratory trial with no prospective sample size calculation. The number of tested implants and treated patients was selected empirically.

## Results

Within the observation period, 10 consecutive patients (7 female, 3 male) received 36 implants with hydrophilic surface. At implant insertion the patients were 23 to 72 years old, the mean age was 50.9 years.

35 implants were inserted in regions with bone quality D3 and D4 and were included in the study (one implant in site with bone quality D2 was excluded). The overview of the inserted implants is shown in Table 1. Study implants were inserted in six patients with bone quality D3 and five patients in bone quality D4. One patient received implants in areas of bone quality D3 and D4. In terms of bone quality, 20 implants were inserted in bone quality D3 and 15 in D4.

**Table 1:** Summary of Inserted ELEMENT® INICELL® implants

**Length (mm)**	**PF Ø mm**		
	**4.0**	**4.5**	**5.0**
**8.0**	3	5	5
**9.5**	1	3	1
**11.0**	0	2	3
**12.5**	0	4	7
**14.0**	1	0	0

16 implants (45.7%) were placed in the maxilla and 19 implants (54.3%) in the mandible. 15 implants were inserted in 5 patients following sinus floor elevation. 12 implants were placed in 3 additional patients with simultaneous bone augmentation procedures.

31 implants (88.6%) were released for loading after 8 weeks and 2 implants (5.7%) after 12 weeks. Two implants (5.3%) were lost before release for loading. In one patient an implant inserted after sinus floor elevation had to be explanted four weeks after implant insertion due to a broken Osstell®-Smartpeg while mounted in the implant body. Due to this technical complication, this implant was excluded from the overall success calculation.

The second implant was removed in a female patient 12 weeks post implantation. During the implant osseointegration progress, wound healing disturbances such as dehiscence of the peri-implant soft tissue and bone exposure became apparent. Four years before the surgery this patient had a partial mouth floor resection with subsequent radiation therapy. The dehiscence healed after the implant removal without any further complications.

**Figure 1:**
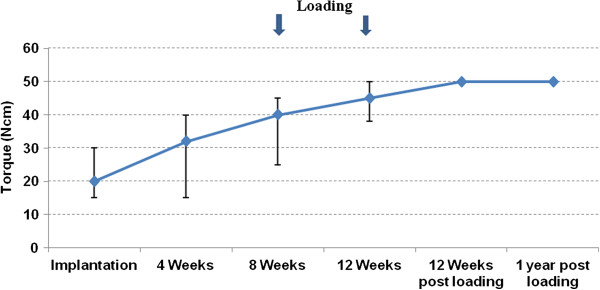
**Torque analysis.** The error bars represent minimum and maximum measured values.

At the time of implantation the average insertion torque was 23 ± 3 Ncm. The tactile resistance at implant uncovering after four weeks was 32 ± 6 Ncm, increasing to 40 ± 5 Ncm after eight weeks. At 12 weeks it reached 45 ± 3 Ncm, whereas 12 weeks and 1 year after loading the maximal measurable value of 50 Ncm was measured for all implants (Table 2, Figure 1).

**Table 2:** Torque analysis

**Torque value**	**Implantation**	**4 WKS**	**8 WKS**	**12 WKS**	**12 WKS post loading**	**1 year post loading**
Mean (Ncm)	21.3	32.1	39.6	45	50	50
Min	15	15	25	38	50	50
Max	30	40	45	50	50	50
Standard deviation	3.3	5.7	5.1	---	---	---
CI (95%)	20.1-22.5	30.0-34.2	37.7-41.5			

WKS = Weeks.

**Figure 2:**
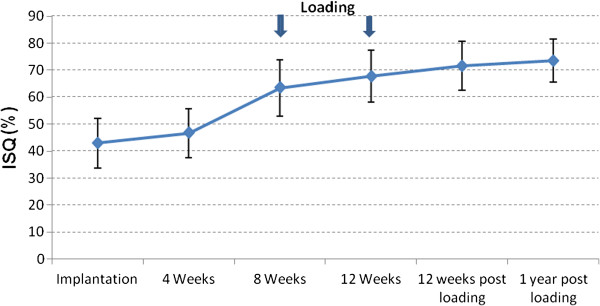
**ISQ analysis.** The error bars represent minimum and maximum measured values.

The mean ISQ value immediately after implantation was 43 ± 9 and four weeks later it increased to 47 ± 9. A distinct rise was recorded in the second measurement interval between 4 and 8 weeks. After 8 weeks, a mean ISQ value of 63 ± 10 was found, increasing to 68 ± 10 after 12 weeks for the non-loaded implants. 12 weeks after loading, ISQ value of 72 ± 9 was found, and remained more or less constant with 73 ± 8 until the end of the follow up period Figure 2, (Table 3).

**Table 3:** ISQ analysis

**ISQ**	**Implantation**	**4 WKS**	**8 WKS**	**12 WKS**	**12 WKS post loading**	**1 year post loading**
Mean (%)	43	47	63	68	72	73
Median	43	47	64	66	74	76
SD	9	9	10	10	9	8
CI (95%)	40-46	41-50	59-67	64-72	69-75	70-76
Min	26	28	39	51	54	58
Max	56	58	82	83	84	84

WKS = Weeks.

**Figure 3:**
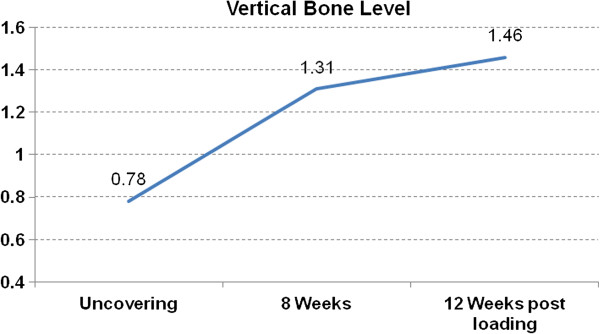
Distance of implant shoulder to bone.

**Figure 4:**
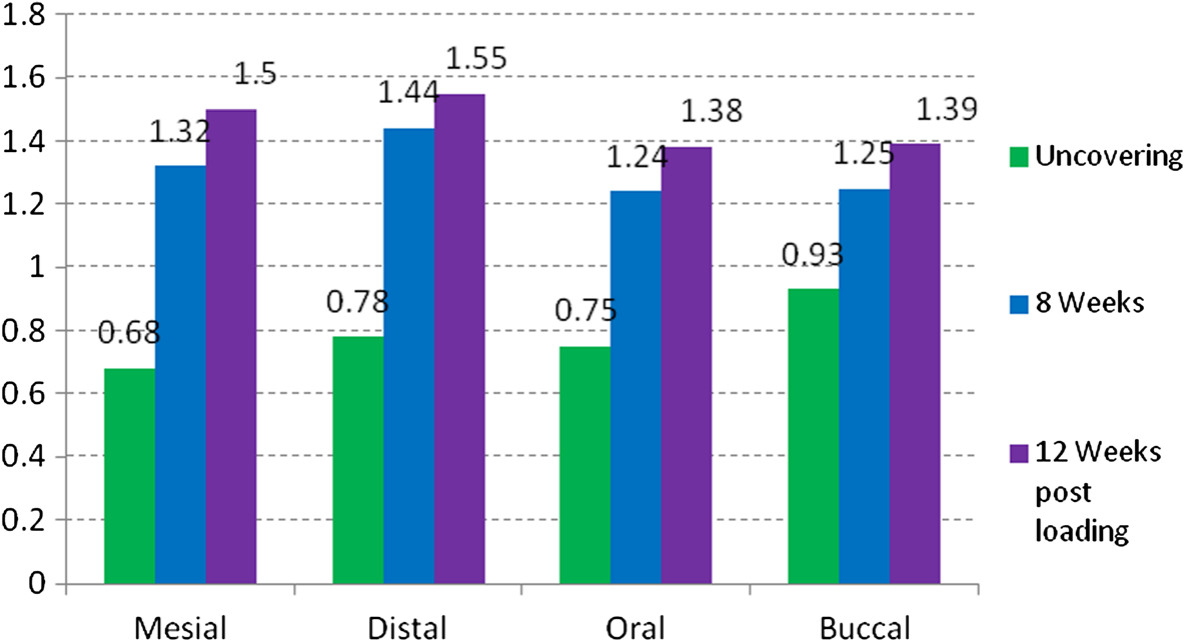
Vertical bone level related to implant surface.

The evaluation of the vertical bone level (Table 4, Figure 3) was measured using a calibrated periodontal probe using the implant shoulder as starting point. At implant installation all implants were inserted epicrestally with the implant shoulder on the surrounding bone level. At implant uncovering the total value of pocket depth was 0.78 ±0.49 mm. After eight weeks an average of 1.31 ± 0.55 mm was found. 12 weeks after release of the implants for prosthetic loading, the average distance from implant shoulder to marginal bone was 1.46 ± 0.7 mm. It is to be noted that the bone loss was most prominent in mesial and distal regions with a mean of 1.5 mm mesially, and a mean of 1.55 mm distally (Table 4, Figure 4).

**Table 4:** Distance of implant shoulder to bone

	**Vertical bone level (mm)**	**Standard-deviation (SD)**	**95% Confidence interval**
4 weeks	0.78	0.49	0.0 - 1.59
8 weeks	1.31	0.55	0.41 - 2.21
12 weeks post loading	1.46	0.7	0.31 - 2.61

## Discussion

The objective of the presented prospective clinical trial was the investigation of the behavior of implants with chemically modified surface using an early loading protocol in bone with reduced density. It was found that in most cases (89%) even in D3 and D4 bone an early loading protocol with prosthetic rehabilitation of hydrophilic implants after 8 weeks could be successfully performed.

The chemical modification of the used implants is based on conditioning of the surface with OH-ions. Hydroxylated titanium surfaces possess higher surface free energy and hydrophilicity [20] leading to increased production of osteogenic factors such as osteocalcin and growth factors [10]. In a pilot study in dogs, Schwarz et al. [21] found a significantly stronger proliferation of vascular structures as well as an increased osteocalcin activity and improved bone building processes for chemically modified, hydroxylated surfaces as compared to conventional surfaces.

A faster bone apposition rate was found particularly in the early healing phase of implant osseointegration, notably in the time between two to four weeks [3,22]. After two to four weeks of healing, BIC of implants with hydrophilic surface was significantly higher when compared to the control group containing titanium implants with a conventional surface. Buser et al. [3] have shown that BIC was 60% higher when using hydrophilic surfaces compared to the control group. After 8 weeks, however, the BIC values did not show statistically significant differences. A similar behavior was also shown with respect to bone density [21,23]. The change in peri-implant bone density or of bone-to-implant contact rate are indicators of the osseointegrative properties of the implants. In addition to primary stability [24,25], it is the extent of osseointegration during the healing phase that determines the time course of implant loading with prosthetic suprastructures [26].

The determination of primary stability of the 35 implants that were inserted in areas with reduced bone density or reduced bone quality was done by measurement of the insertion torque. The measurement of the insertion torque for quantitative determination of implant primary stability is scientifically established [27,28]. For the determination of bone quality, initially three-dimensional X-ray images obtained by CBCT were made. An exact pre-implantological measurement of the bone mineral density can be done using quantitative computer tomography [29]. The latest studies have shown a correlation between the voxel values of CBCT and the degree of mineralization of the bone obtained by determination of the density by the computer tomography [30]. In a study by Isoda et al. [31] a significant relationship was shown between the estimated bone density determined by CBCT and primary stability of the implants. The primary stability was determined by measurement of resonance frequency analysis (RFA) in addition to measurement of bone density and also compared to the insertion torque [31]. The CBCT used in the presented study served the purpose of visual estimation of the bone quality according to Misch [18], as well as an estimation of the thickness of the compacta in relation to the spongiosa, similar to Lekholm and Zarb [19]. Misch [18] divided the bone into four different quality classes (D1 to D4) whereas each of the four classes were topologically matched and described according to their implantological value. Bone type D3 and D4 was characterized through a thin, porous compacta and a coarse-meshed spongiosa comparable to bone Class 3 and 4 according to Lekholm and Zarb [19]. Bone of this quality (or density) is found predominantly in upper jaw regions, whereas the tuber area and the augmented regions typically show D3 and D4 characteristics [19].

In the presented investigation a total of 27 implants have been inserted in augmented areas. The augmentation occurred in part simultaneously with the implantation.

To determine the primary stability of the implants, tactile resistance was measured using the MONO® torque ratchet i.e. by a careful assessment of the torque momentum in the “OUT” position. Correlations between implant insertion torques and the density of bone mineralization have been demonstrated [32]. In an investigation of two different implant systems by Rabel et al. [33], mean torque values of 22.1 Ncm were measured in the maxillary regions. Furthermore mean torque values of 25.5 Ncm were determined in D2 and D3 bones as well as 24.3 Ncm for implants in augmented bone.

In the present study the mean value of the insertion torque at the time point of implantation was 20 Ncm (minimum 15 Ncm, maximum 35 Ncm). For the assessment of implant stability, in addition to insertion torque evaluation, the measurement of RFA has been established [34-37]. The advantages of RFA measurement are firstly its non-invasiveness and secondly the high reproducibility of its investigation. That is the reason why the measurement of the ISQ is used to check the course of osseointegration with particular focus on the time point of prosthetic loading feasibility [38,39]. However it has to be mentioned that also RFA analysis has a risk of complications. In the present study, a fracture of an Osstell®-Smartpeg led to a necessity of implant removal. Although the rate of Smartpeg fractures is low, study and routine patients have to be informed and give their informed consent when using this kind of implant stability measurement devices.

Jimenez et al. [26] have shown a markedly higher implant success rate for implants that were loaded when their ISQ value was >50. Success rates between 96.75 and 98.1% were demonstrated within several investigations aimed at immediate loading of dental implants when loading was done at ISQ values > 60 [24,25,40]. Ostman et al. [41] have found a success rate of 98.4% for implants in partially dentate lower jaw. The tested implants had to show at insertion an ISQ value >60 and an insertion torque of more than 30 Ncm. In the presented study the decision concerning the time point of occlusal loading was made 8 weeks after implantation. Implants that withstood the measurement of tactile resistance (ratchet in position “OUT”) of at least 35 Ncm were released for loading. This was the case for 31 of 35 implants. The mean value of ISQ was 63 (minimum 39, maximum 82). The two remaining implants were loaded 4 weeks later as the obtained result after eight weeks was <35 Ncm. ISQ measurement took place at the time of implantation as well as after 4, 8 or 12 weeks and 12 weeks and one year after loading. At the time of implant insertion the mean ISQ value of all implants was 43 ± 9, initially this increased moderately until implant uncovering after four weeks to 47 ± 9. Between implant uncovering and the first follow-up visit after eight weeks a more marked increase of ISQ values occurred (63 ± 10). This might be an effect of the ongoing osseointegration, probably further induced by the moderate initial loading of the implant based on the transgingival position of the gingiva former. During the third follow up 12 weeks after loading the increase of ISQ mean values was again less pronounced: 68 (SD ± 10) or 72 (SD ± 9), respectively.

The pattern of ISQ changes can be explained with reference to an investigation by Oates et al. [12]. The authors tested the implant stability by means of RFA for implants with chemically modified, hydrophilic surface. In line with the present study, ISQ values were determined using the Osstell® device. Implants showed an initial decrease of the ISQ values within the first two 2 weeks. After six weeks, the ISQ values got back, closer to starting values. An additional increase took place until week 12 although this was less steep then after 6 weeks.

A curve with similar pattern of ISQ changes after insertion of implants with chemically modified surface has been published by Schätzle et al. [42]. In their study the authors observed an initial decrease of ISQ values followed by a marked increase only 28 days later. Bornstein et al. [22] on the other hand reported a steady increase of ISQ values within a 6 months follow up study, from a mean of 74.3 at the time of implantation to 83.8 after 26 weeks, for early loaded implants with a chemically modified surface.

In addition to the analysis of implant stability, an additional emphasis of the presented study was the assessment of the marginal bone level in the area of implant shoulder. At implant uncovering the peri-implant bone status was measured using a calibrated parodontal probe at mesial, distal, buccal and oral sites. The average of the mean distances was 0.78 ± 0.49 mm at the time of implant uncovering. After 6 months this mean distance was 1.46 ± 0.7 mm. A loss of marginal periimplant bone of 0.68 mm after implant uncovering is comparable with the results by Ostman er al. [41], analyzing the marginal bone level at 257 implants.

Altogether 31 of 35 implants that were inserted into bone Class 3 and 4 were loaded 8 weeks after successful implantation and 2 implants after 12 weeks. This reveals an implant survival rate of 94.3%. Excluding the implant that had to be removed due to Osstell® technical complications, a success rate of even 97,1% might be calculated. One implant had to be removed in a female patient due to bone dehiscence. This 64 year old female patient has previously received radiation therapy. She was treated by an extensive vestibuloplasty in combination with implantation. In addition a mobilization of the mouth floor was done to free her tongue. A minor perforation of the periosteum has occurred in the area of one of the implants and successively the wound healing was disturbed. Although it was reported that hydrophilic implants show also very good survival rates in irradiated patients [4,43], the implant was considered non-successful and was removed using a ratchet. After implant removal the soft tissue regenerated completely.

The influence of structured and chemically modified implant surfaces on the osseointegration is a topic of multiple scientific studies [44-46]. Due to various surface modifications a shorter healing time of dental implants can be achieved and as a result faster loading with prosthetic suprastructures is feasible [12]. Considering reduced implant healing time several manufacturers focus on a chemical modification of the enossal surface [21,47]. The chemical modification lends the enossal surface the improved hydrophilicity. Based on the experience with implant systems with similar surfaces it can be reasonably expected that this surface type will lead to an improved and faster bone healing [3,21].

At the final follow up all implants that were released for loading have been stable and in situ. The analysis of the presented results indicated good osseointegrative properties of the implants with the novel INICELL® surface. Overall, the data evaluated indicate that the hydrophilic implants investigated show good osseointegrative properties, even in bone quality Class 3 and 4 [18] or D3 and D4 [19]. Consequently early loading of the implants, that were inserted in this compromised bone quality, was possible. However, further studies with a larger number of patients longer follow up periods are need to draw statistically relevant conclusions about shorter healing time of implants with chemically modified enossal surface inserted into bone with reduced density.

## Conclusions

Within the limitations of this prospective clinical trial it was concluded that (i) chemically modified, hydrophilic implants support early osseointegration even in D3 and D4 bone (ii) shortening the healing period in patients with low density bone seems to be feasible after individual evaluation of implant stability. Further studies with higher implant numbers and longer observation periods are necessary to allow for general recommendations regarding shorter healing periods for hydrophilic implants in D3 and D4 bone.

## Abbreviations

CBCT: Cone beam computed tomography; ISQ: Implant stability quotient (Osstell); Ncm: Newtoncentimeter; OPG: Orthopantomogram.

## Competing interests

In the past 5 years Dr. Held, Dr. Rohner or Dr. Rothamel have not received reimbursements, fees, funding, or salary from Thommen Medical AG. Thommen Medical AG will reimburse the article-processing charge. Dr. Held, Dr. Rohner or Dr. Rothamel are not shareholders of Thommen Medical AG. None of the authors neither holds nor is applying any patents relating to the contents of this manuscript.

## Authors’ contributions

Dr. U Held and Dr. D Rohner are co-authors of the Study protocol. They were essential in obtaining the Ethics Committee approval, study conduct and evaluation. Dr. D Rothamel made a significant intellectual contribution to result evaluation and paper compilation. All authors read and approved the final manuscript.
